# Supplementary figures and images for: Identification of Susceptibility Genes for Fusarium oxysporum in Cucumber via Comparative Proteomic Analysis
Source: Genes (Basel). 2021 Nov 10;12(11):1781. doi: 10.3390/genes12111781 (PMC8623666; doi:10.3390/genes12111781)

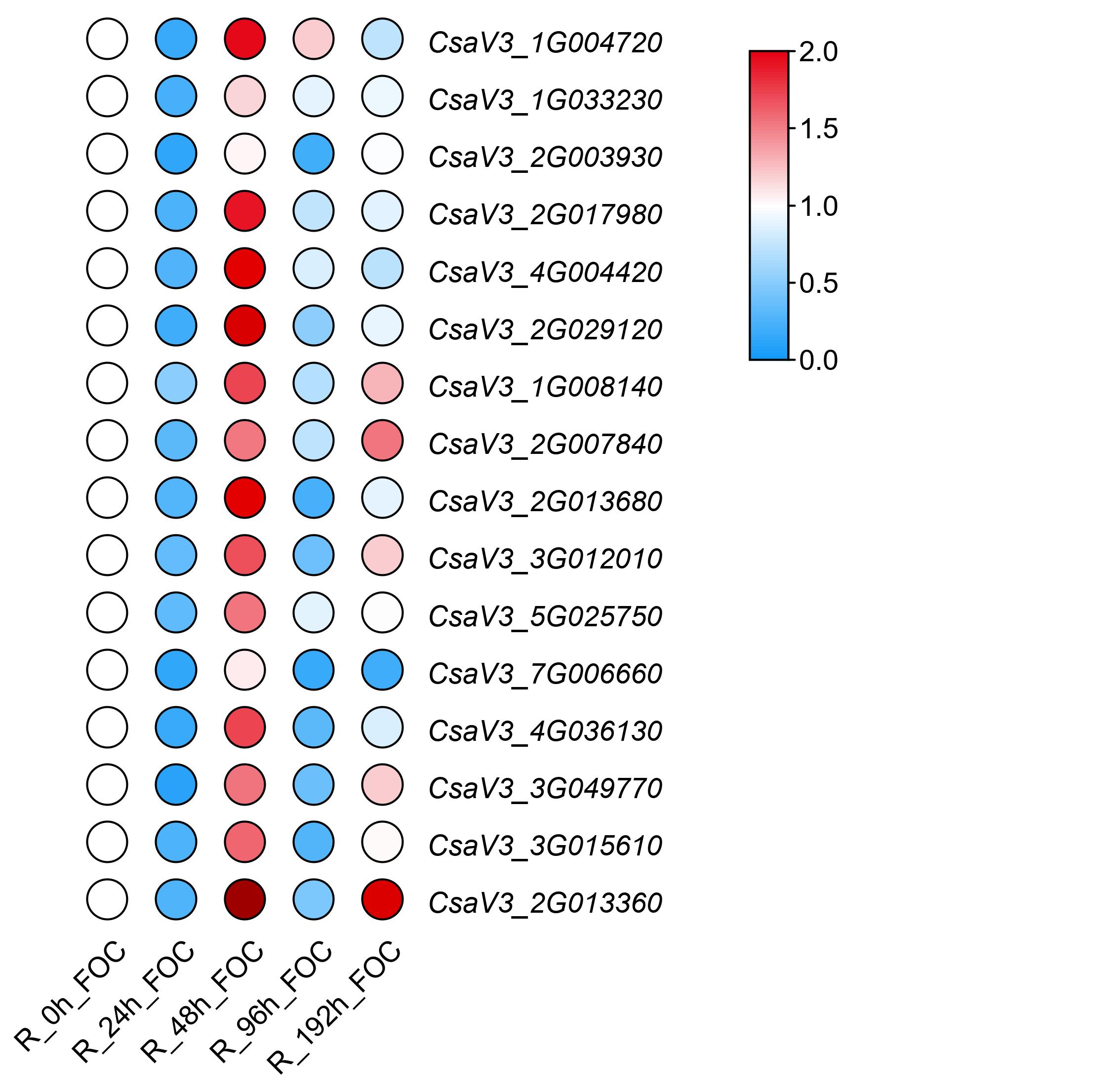

Supplement: Supplementary file 1 [file genes-12-01781-s001.zip › Additional file 4 Figure S1 Expression of candidate genes in Rijiecheng .jpg]
